# A mathematical model of *in vitro* hepatocellular cholesterol and lipoprotein metabolism for hyperlipidemia therapy

**DOI:** 10.1371/journal.pone.0264903

**Published:** 2022-06-03

**Authors:** Yuri Efremov, Anastasia Ermolaeva, Georgiy Vladimirov, Susanna Gordleeva, Andrey Svistunov, Alexey Zaikin, Peter Timashev

**Affiliations:** 1 Institute for Regenerative Medicine, Sechenov First Moscow State Medical University (Sechenov University), Moscow, Russia; 2 World-Class Research Center “Digital Biodesign and Personalized Healthcare”, Sechenov University, Moscow, Russia; 3 Lobachevsky State University of Nizhny Novgorod, Nizhny Novgorod, Russia; 4 Neuroscience and Cognitive Technology Laboratory, Center for Technologies in Robotics and Mechatronics Components, Innopolis University, Innopolis, Russia; 5 Sechenov First Moscow State Medical University (Sechenov University), Moscow, Russia; 6 Department of Mathematics, University College London, London, United Kingdom; 7 Institute for Women’s Health, University College London, London, United Kingdom; 8 Centre for Analysis of Complex Systems, Sechenov University, Moscow, Russia; 9 Chemistry Department, Lomonosov Moscow State University, Moscow, Russia; Universidade de Vigo, SPAIN

## Abstract

Cardiovascular diseases associated with high cholesterol (hypercholesterolemia) and low-density lipoproteins (LDL) levels are significant contributors to total mortality in developing and developed countries. Mathematical modeling of LDL metabolism is an important step in the development of drugs for hypercholesterolemia. The aim of this work was to develop and to analyze an integrated mathematical model of cholesterol metabolism in liver cells and its interaction with two types of drugs, statins and PCSK9 inhibitors. The model consisted of 21 ordinary differential equations (ODE) describing cholesterol biosynthesis and lipoprotein endocytosis in liver cells *in vitro*. The model was tested for its ability to mimic known biochemical effects of familial hypercholesterolemia, statin therapy, and PCSK9 inhibitors. The model qualitatively reproduced the well-known biology of cholesterol regulation, which confirms its potential for minimizing cellular research in initial testing of new drugs for cardiology.

## Introduction

Atherosclerotic cardiovascular diseases (ACVD) are the largest cause of morbidity and premature death in developed countries [1–3]. Hypercholesterolemia, high levels of circulating plasma low-density lipoprotein cholesterol (LDL-C), is considered as one of the main risk factors for ACVD progression [4, 5]. Cholesterol is an important component in the functioning of individual cells and a whole organism, and liver is primarily responsible for cholesterol metabolism and its removal from the body via the formation of bile.

Low-density lipoproteins (LDL) and very-low-density lipoproteins (VLDL) are carrying cholesterol through the bloodstream until they are removed from circulation by liver cells via so-called receptor-mediated endocytosis (RME). The rate of lipoprotein uptake is regulated by the number of available LDL receptors (LDLRs) on the cell surface [6]. LDLR synthesis is regulated by intracellular cholesterol level that depends both on RME rate and the activity of intracellular cholesterol synthesis. A specific regulatory protein, proprotein convertase subtilisin kexin type 9 (PCSK9), inhibits the cell uptake of LDL-cholesterol from plasma by directly interacting with LDLR, which leads to a degradation of LDLR [7, 8]. Therefore, two strategies among others were suggested against hypercholesterolemia: inhibition of intracellular cholesterol biosynthesis by statins and a variety of anti-PCSK9 therapeutic modalities [4, 5, 9–11]. Statins competitively bind to 3-hydroxy-3-methyl-glutaryl-coenzyme A reductase (HMGCR) and block the associated synthesis pathway [10]. Anti-PCSK9 therapeutic modalities include antibodies, small molecules or peptides, and siRNA [9, 12–15].

The field of the mathematical modeling of lipoprotein metabolism is intensively growing, aiming to reproduce the known effects of hypercholesterolemia and different treatments against it [16–23]. Eventually, such a model will help researchers in both the development and quantitative benchmarking of different pharmacological approaches. Here, based on the previously developed model of integrated cholesterol biosynthesis [18], we aimed to combine in a single model both statin and anti-PCSK9 therapies. The model is formulated in an *in vitro* context, which is an important step in testing any potential drug, especially considering recent developments in experimental cell-based models [24–28].

## Materials and methods

### Model formulation

Our work is based on the integrated mathematical model of cellular cholesterol biosynthesis and lipoprotein metabolism developed in [18]. The model consists of three main compartments. The first compartment is the cell nucleus, where genetic regulation of HMGCR and LDLR occurs. In our model we added genetic regulation of PCSK9 as well. The second compartment is the cell cytoplasm surrounded by the cell membrane, where all processes related to VLDL and LDL binding and breakdown, cell receptor, and cholesterol regulation take place. The third compartment is the extracellular space containing sources of VLDL and LDL. Here we also modified the model to account for the addition of PCSK9, anti-PCSK9 antibodies or small molecules, and statins. Out model is formulated for a specific *in vitro* cell experiment, thus it implies the addition of components to the cell medium at selected time points.

**Fig 1:**
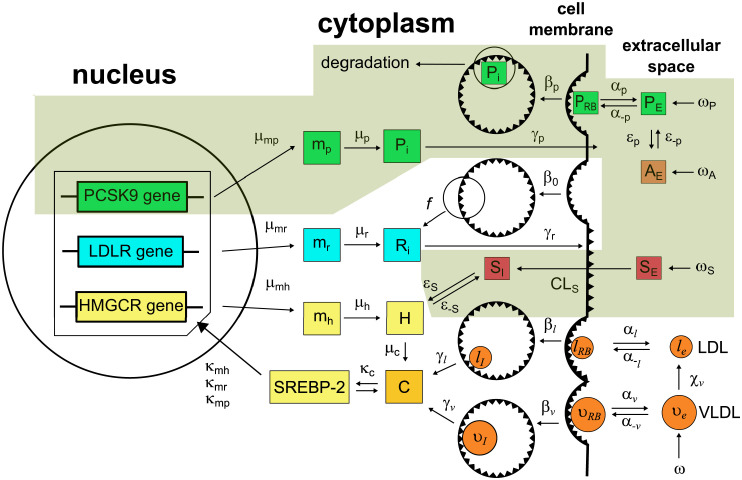
The model of cholesterol-lipoprotein metabolism and interaction with lipid-lowering agents in a hepatocyte. Inside the cell (nucleus and cytoplasm), transcription of HMGCR (H), LDLR (R) and PCSK9 (P) mRNA (at rates *μ*_*mh*_, *μ*_*mr*_, and *μ*_*mp*_, respectively) is activated by SREBP-2 (*κ*_*mh*_, *κ*_*mr*_, *κ*_*mp*_), and the synthesis occurs at rates *μ*_*h*_, *μ*_*r*_, and *μ*_*p*_, respectively. Cholesterol (C) is produced by the activity of HMGCR (*μ*_*c*_) and negatively regulates SREBP-2 (*κ*_*c*_). LDLR (*γ*_*r*_) and PCSK9 (*γ*_*p*_) are transported to the cell membrane and extracellular space, respectively. In the extracellular space, VLDL, LDL and PCSK9 bind/unbind to LDLR on the cell surface (*α*_*V*_, *α*_*l*_, *α*_*P*_). LDLRs, both bound and unbound to lipoproteins or PCSK9 are endocytosed, and part of LDLRs is recycled in the intracellular space. Cholesterol is extracted from internalised lipoproteins (*γ*_*V*_ and *γ*_*l*_). In the extracellular space, interaction of PCSK9 with anti-PCSK9 agents (A, ${\bar{\varepsilon }}_{p}$) also occurs. Statins (S) are internalised into the cell (clearance *CL*_*S*_) and inhibit HMGCR (*ε*_*S*_). The degradation pathways are not shown for better visualization. The scheme is created on the basis of the scheme presented in [18]. The newly introduced pathways are highlighted.

The model in [18] was formulated by applying the Law of Mass Action and was further reduced by the application of the quasi-steady-state approximation and conservation laws. Here we have utilized most of the parameters used in [18] (S1 Appendix). Newly introduced parameters are presented in Table 1. The parameters were derived from the experimental literature, wherever available, or based on the underlying biological principles and plausible physiologic limits of the model outcome. The schematic representation of the model is presented in Fig 1. Below we describe the model and our additions to it.

**Table 1:** Newly introduced model parameters. Molec. denotes molecules.

Parameter	Description	Dimensional Value	Units	Reference
${\bar{\mu }}_{mp}^{*}$	Rate of PCSK9 mRNA transcription	1 × 10^6^	$\frac{molec.}{mL\;s}$	This study
${\bar{\mu }}_{p}$	Rate of PCSK9 translation	1 × 10^−1^	$\frac{1}{s}$	This study
${\bar{\delta }}_{mp}$	Rate of PCSK9 mRNA degradation	4.48 × 10^−5^	$\frac{1}{s}$	[18]
*x*_*p*_	Number of binding sites for SREBP-2 on PCSK9 gene	1	-	This study
${\bar{k}}_{mp}$	PCSK9 gene-SREBP-2 binding affinity	8.21 × 10^16^	$\frac{molec.}{mL}$	[18]
${\bar{\gamma }}_{p}$	Rate of PCSK9 transport to the extracellular space	1 × 10^−2^	$\frac{1}{s}$	This study
${\bar{\delta }}_{p}$	Rate of intracellular PCSK9 degradation	0	$\frac{1}{s}$	This study
${\bar{\alpha }}_{p}$	Rate of PCSK9-receptor binding	1 × 10^−17^	$\frac{mL}{molec.\;s}$	[8]
${\bar{\alpha }}_{-p}$	Rate of PCSK9-receptor unbinding	1 × 10^−4^	$\frac{1}{s}$	[8]
*M*_*p*_	Receptors covered by bound PCSK9	1	-	This study
${\bar{\beta }}_{p}$	Rate of PCSK9 internalisation	2.7 × 10^−3^	$\frac{1}{s}$	[18]
${\bar{\varepsilon }}_{S}$	Rate of Statin-HMGCR binding	1 × 10^−14^	$\frac{mL}{molec.\;s}$	[31]
${\bar{\varepsilon }}_{-S}$	Rate of Statin-HMGCR unbinding	1 × 10^−2^	$\frac{1}{s}$	[31]
$\bar{C}L_{S}$	Cell uptake clearance of statins	0.01	$\frac{mL}{s}$	[32]
${\bar{\varepsilon }}_{p}$	Rate of antibody-PCSK9 binding	1 × 10^−14^	$\frac{mL}{molec.\;s}$	[33]
${\bar{\varepsilon }}_{-p}$	Rate of antibody-PCSK9 unbinding	1 × 10^−3^	$\frac{1}{s}$	[33]

The first group of Eqs (1)–(6) describes transcription, translation, and mRNA degradation processes (nucleus—cytoplasm compartments) of HMGCR, LDLR, and PCSK9. The PCSK9 intracellular synthesis pathway was added here by analogy with the presented in the model HMGCR and LDLR pathways. Importantly, transcription of all of these proteins is regulated by the same transcription factor, sterol regulatory element-binding protein 2 (SREBP-2), Eqs (1)–(3) [29]. SREBP-2 is blocked when the cellular levels of cholesterol are high, which is accounted for through the SREBP-Cholesterol dissociation constant, ${\bar{k}}_{c}$. Parameter $\bar{\kappa }$ is the gene binding affinity; *x*_*c*_ is the number of cholesterol molecules to inactivate SREBP-2; *x*_*h*_, *x*_*r*_, *x*_*p*_ correspond to the number of binding sites for SREBP-2 on HMGCR, LDLR and PCSK9 genes, respectively; and $\bar{\delta }$ is the rate of mRNA degradation.

$$\begin{array}{c} J\frac{d{\bar{m}}_{h}}{d\bar{t}}=\frac{{\bar{\mu }}_{mh}^{*}}{1+{\left(\frac{{\bar{\kappa }}_{mh}\left(1+{\left(\frac{\bar{c}}{{\bar{k}}_{c}}\right)}^{x_{c}}\right)}{{\bar{s}}_{0}}\right)}^{x_{h}}}-{\bar{\delta }}_{mh}{\bar{m}}_{h}; \end{array}$$ (1)

$$\begin{array}{c} J\frac{d{\bar{m}}_{r}}{d\bar{t}}=\frac{{\bar{\mu }}_{mr}^{*}}{1+{\left(\frac{{\bar{\kappa }}_{mr}\left(1+{\left(\frac{\bar{c}}{{\bar{k}}_{c}}\right)}^{x_{c}}\right)}{{\bar{s}}_{0}}\right)}^{x_{r}}}-{\bar{\delta }}_{mr}{\bar{m}}_{r}; \end{array}$$ (2)

$$\begin{array}{c} J\frac{d{\bar{m}}_{p}}{d\bar{t}}=\frac{{\bar{\mu }}_{mp}^{*}}{1+{\left(\frac{{\bar{\kappa }}_{mp}\left(1+{\left(\frac{\bar{c}}{{\bar{k}}_{c}}\right)}^{x_{c}}\right)}{{\bar{s}}_{0}}\right)}^{x_{p}}}-{\bar{\delta }}_{mp}{\bar{m}}_{p}. \end{array}$$ (3)

Eqs (4)–(6) describe the synthesis of HMGCR, PCSK9, and LDLR by the cell. The equations also account for intracellular protein degradation ($\bar{\delta }$), transport to the membrane (${\bar{\gamma }}_{r}$ for LDLR), or to the extracellular space (${\bar{\gamma }}_{p}$) as for the newly introduced PCSK9. The equation for HMGCR describes interaction with statins ($\bar{S}$, see below). The equation for LDLR also contains the members describing the recycling of LDLR from empty and containing LDL or VLDL vesicles. We also consider here that there is no recycling of LDLR from vesicles with PCSK9.

$$\begin{array}{c} \frac{d\bar{h}}{d\bar{t}}={\bar{\mu }}_{h}{\bar{m}}_{h}-{\bar{\delta }}_{h}\bar{h}-{\bar{\varepsilon }}_{S}{\bar{S}}_{i}\bar{h}+{\bar{\varepsilon }}_{-S}{\bar{S}}_{ih}; \end{array}$$ (4)

$$\begin{array}{c} \frac{d{\bar{p}}_{I}}{d\bar{t}}={\bar{\mu }}_{p}{\bar{m}}_{p}-{\bar{\gamma }}_{p}{\bar{p}}_{I}-{\bar{\delta }}_{p}{\bar{p}}_{I}; \end{array}$$ (5)

$$\begin{array}{c} \frac{d{\bar{r}}_{I}}{d\bar{t}}={\bar{\mu }}_{r}{\bar{m}}_{r}-{\bar{\gamma }}_{r}{\bar{r}}_{I}+fP{\bar{\beta }}_{0}{\bar{r}}_{f}^{P}+fM_{l}{\bar{\beta }}_{l}\left(\frac{{\bar{r}}_{f0}{\bar{l}}_{RB}}{{\bar{r}}_{f0}-{\bar{r}}_{f}}\right)+fM_{v}{\bar{\beta }}_{v}\left(\frac{{\bar{r}}_{f0}{\bar{v}}_{RB}}{{\bar{r}}_{f0}-{\bar{r}}_{f}}\right). \end{array}$$ (6)

Eq 7 describes the kinetics of free unbound receptors ${\bar{r}}_{f}$ on the plasma membrane. It is recycled from the internalized receptor pool, and depleted by internalization and binding with LDL ($\bar{l}$), VLDL ($\bar{v}$), or PCSK9 ($\bar{p}$). The number of receptors associated with a single molecule is accounted with parameters *M*_*l*_, *M*_*v*_, *M*_*p*_. The binding/unbinding rates are determined by *α*/*α*_−_, and *β* describes the internalisation rates:

$$\begin{array}{ll} \frac{d{\bar{r}}_{f}}{d\bar{t}}={\bar{\gamma }}_{r}{\bar{r}}_{I}-P{\bar{\beta }}_{0}{\bar{r}}_{f}^{P} & -M_{l}{\bar{\beta }}_{l}(\frac{{\bar{r}}_{f}{\bar{l}}_{RB}}{{\bar{r}}_{f0}-{\bar{r}}_{f}})-M_{l}{\bar{\alpha }}_{l}{\bar{r}}_{f}{\bar{l}}_{E}+M_{l}{\bar{\alpha }}_{-l}{\bar{l}}_{RB}-\\ & -M_{v}{\bar{\beta }}_{v}(\frac{{\bar{r}}_{f}{\bar{v}}_{RB}}{{\bar{r}}_{f0}-{\bar{r}}_{f}})-M_{v}{\bar{\alpha }}_{v}{\bar{r}}_{f}{\bar{v}}_{E}+M_{v}{\bar{\alpha }}_{-v}{\bar{v}}_{RB}-\\ & -M_{p}{\bar{\beta }}_{p}(\frac{{\bar{r}}_{f}{\bar{p}}_{RB}}{{\bar{r}}_{f0}-{\bar{r}}_{f}})-M_{p}{\bar{\alpha }}_{p}{\bar{r}}_{f}{\bar{p}}_{E}+M_{p}{\bar{\alpha }}_{-p}{\bar{p}}_{RB}. \end{array}$$ (7)

Eqs 8–13 describe the level of extracellular and internalized LDL and VLDL. These equations detail the association of extracellular LDL and VLDL to receptors and the delipidation of VLDL to LDL. The indexes *E*, *I*, and *RB* refer to extracellular, intracellular, and receptor-bound molecules, respectively. As in the original model [18], a constant source of VLDL, $\bar{\omega }$, is presented in our model. Internalization of LDL and VLDL occurs via endocytosis and bound pits:

$$\begin{array}{c} W\frac{d{\bar{l}}_{E}}{d\bar{t}}=-{\bar{\alpha }}_{l}{\bar{r}}_{f}{\bar{l}}_{E}+{\bar{\alpha }}_{-l}{\bar{l}}_{RB}+W{\bar{\chi }}_{v}{\bar{v}}_{E}; \end{array}$$ (8)

$$\begin{array}{c} \frac{d{\bar{l}}_{RB}}{d\bar{t}}={\bar{\alpha }}_{l}{\bar{r}}_{f}{\bar{l}}_{E}-{\bar{\alpha }}_{-l}{\bar{l}}_{RB}-{\bar{\beta }}_{l}{\bar{l}}_{RB}; \end{array}$$ (9)

$$\begin{array}{c} \frac{d{\bar{l}}_{I}}{d\bar{t}}={\bar{\beta }}_{l}{\bar{l}}_{RB}-{\bar{\gamma }}_{l}{\bar{l}}_{I}; \end{array}$$ (10)

$$\begin{array}{c} W\frac{d{\bar{v}}_{E}}{d\bar{t}}=-{\bar{\alpha }}_{v}{\bar{r}}_{f}{\bar{v}}_{E}+{\bar{\alpha }}_{-v}{\bar{v}}_{RB}-W{\bar{\chi }}_{v}{\bar{v}}_{E}+W\bar{\omega }; \end{array}$$ (11)

$$\begin{array}{c} \frac{d{\bar{v}}_{RB}}{d\bar{t}}={\bar{\alpha }}_{v}{\bar{r}}_{f}{\bar{v}}_{E}-{\bar{\alpha }}_{-v}{\bar{v}}_{RB}-{\bar{\beta }}_{v}{\bar{v}}_{RB}; \end{array}$$ (12)

$$\begin{array}{c} \frac{d{\bar{v}}_{I}}{d\bar{t}}={\bar{\beta }}_{v}{\bar{v}}_{RB}-{\bar{\gamma }}_{v}{\bar{v}}_{I}. \end{array}$$ (13)

Eq 14 describes the endogenous and exogenous cholesterol regulation, accounting for the effect of cholesterol binding/unbinding to free SREBP-2:

$$\begin{array}{c} \frac{d\bar{c}}{d\bar{t}}=R_{l}^{chol}{\bar{\gamma }}_{l}{\bar{l}}_{I}+R_{v}^{chol}{\bar{\gamma }}_{v}{\bar{v}}_{I}+{\bar{\mu }}_{c}\bar{h}-{\bar{\delta }}_{c}\bar{c}. \end{array}$$ (14)

Eqs 15 and 16 were newly introduced and describe the levels of unbound (${\bar{p}}_{E}$) and receptor-bound (${\bar{p}}_{E}$) extracellular PCSK9, a constant or temporary source of external PCSK9 (${\bar{\omega }}_{P}$) was added as well:

$$\begin{array}{c} W\frac{d{\bar{p}}_{E}}{d\bar{t}}=-{\bar{\alpha }}_{p}{\bar{r}}_{f}{\bar{p}}_{E}+{\bar{\alpha }}_{-p}{\bar{p}}_{RB}+{\bar{\gamma }}_{p}{\bar{p}}_{I}+W{\bar{\omega }}_{P}-{\bar{\varepsilon }}_{p}{\bar{A}}_{E}{\bar{p}}_{E}+{\bar{\varepsilon }}_{-p}{\bar{p}}_{AB}; \end{array}$$ (15)

$$\begin{array}{c} \frac{d{\bar{p}}_{RB}}{d\bar{t}}={\bar{\alpha }}_{p}{\bar{r}}_{f}{\bar{p}}_{E}-{\bar{\alpha }}_{-p}{\bar{p}}_{RB}-{\bar{\beta }}_{p}{\bar{p}}_{RB}. \end{array}$$ (16)

Eqs 17 and 18 were introduced for the interaction of PCSK9 with anti-PCSK9 antibodies (or small molecules), based on the assumption that the binding ratio is 1:1 [19, 30]:

$$\begin{array}{c} W\frac{d{\bar{A}}_{E}}{d\bar{t}}=-{\bar{\varepsilon }}_{p}{\bar{A}}_{E}{\bar{p}}_{E}+{\bar{\varepsilon }}_{-p}{\bar{p}}_{AB}+W{\bar{\omega }}_{A}; \end{array}$$ (17)

$$\begin{array}{c} \frac{d{\bar{p}}_{AB}}{d\bar{t}}={\bar{\varepsilon }}_{p}{\bar{A}}_{E}{\bar{p}}_{E}-{\bar{\varepsilon }}_{-p}{\bar{p}}_{AB}. \end{array}$$ (18)

Eqs 15 and 17 also contain temporary (conditional) or constant sources of PCSK9 and anti-PCSk9 agents (${\bar{\omega }}_{P}$ and ${\bar{\omega }}_{A}$, respectively). Otherwise, the molecules can be added via pre-set initial concentration ${\bar{p}}_{E0}$ in the system.

Eqs 19–21 introduce statins into the model. There is a temporary source of statins (${\bar{\omega }}_{S}$). There are several possible ways of statins uptake by hepatocytes, both passive and active. Here, however, we used the single value of cell uptake clearance $\bar{C}L_{S}$, which is a sum of all possible uptake pathways. Such a value can be taken from and compared with *in vitro* experiments.

$$\begin{array}{c} W\frac{d{\bar{S}}_{E}}{d\bar{t}}=-\bar{C}L_{S}{\bar{S}}_{E}+W{\bar{\omega }}_{S}; \end{array}$$ (19)

$$\begin{array}{c} \frac{d{\bar{S}}_{i}}{d\bar{t}}=\bar{C}L_{S}{\bar{S}}_{E}-{\bar{\varepsilon }}_{S}{\bar{S}}_{i}\bar{h}+{\bar{\varepsilon }}_{-S}{\bar{S}}_{ih}; \end{array}$$ (20)

$$\begin{array}{c} \frac{d{\bar{S}}_{ih}}{d\bar{t}}={\bar{\varepsilon }}_{S}{\bar{S}}_{i}\bar{h}-{\bar{\varepsilon }}_{-S}{\bar{S}}_{ih}. \end{array}$$ (21)

Following the work of [18], the model was non-dimensionalized. The used rescalings are presented in S2 Appendix.

The final system of equations is:

$$\begin{array}{c} J\frac{dm_{h}}{dt}=\frac{\mu_{mh}^{*}}{1+{\left(\kappa_{mh}\left(1+{\left(\frac{c}{k_{c}}\right)}^{x_{c}}\right)\right)}^{x_{h}}}-\delta_{mh}m_{h}; \end{array}$$ (22)

$$\begin{array}{c} J\frac{dm_{r}}{dt}=\frac{\mu_{mr}^{*}}{1+{\left(\kappa_{mr}\left(1+{\left(\frac{c}{k_{c}}\right)}^{x_{c}}\right)\right)}^{x_{r}}}-\delta_{mr}m_{r}; \end{array}$$ (23)

$$\begin{array}{c} J\frac{dm_{p}}{dt}=\frac{\mu_{mp}^{*}}{1+{\left(\kappa_{mp}\left(1+{\left(\frac{c}{k_{c}}\right)}^{x_{c}}\right)\right)}^{x_{p}}}-\delta_{mp}m_{p}; \end{array}$$ (24)

$$\begin{array}{c} \frac{dh}{dt}=\mu_{h}m_{h}-\delta_{h}h-\varepsilon_{S}S_{i}h{\bar{S}}_{E0}+\varepsilon_{-S}S_{ih}\frac{{\bar{S}}_{E0}}{{\bar{s}}_{0}}; \end{array}$$ (25)

$$\begin{array}{c} \frac{dp_{I}}{dt}=\mu_{p}m_{p}-\gamma_{p}p_{I}-\delta_{p}p_{I}; \end{array}$$ (26)

$$\begin{array}{c} \frac{dr_{I}}{dt}=\mu_{r}m_{r}-\gamma_{r}r_{I}+f\beta_{0}r_{f}^{P}+f\frac{M_{l}}{v_{l}}\beta_{l}\left(\frac{l_{RB}}{1-r_{f}}\right)+f\frac{M_{v}}{v_{v}}\beta_{v}\left(\frac{v_{RB}}{1-r_{f}}\right); \end{array}$$ (27)

$$\begin{array}{cc} & \begin{array}{cc} \frac{dr_{f}}{dt}=\gamma_{r}r_{I}-\beta_{0}r_{f}^{P} & +\frac{M_{l}}{v_{l}}\left(-\beta_{l}\frac{r_{f}l_{RB}}{1-r_{f}}-\alpha_{l}r_{f}l_{E}+\alpha_{-l}l_{RB}\right)+\\ & +\frac{M_{v}}{v_{v}}\left(-\beta_{v}\frac{r_{f}v_{RB}}{1-r_{f}}-\alpha_{v}r_{f}v_{E}+\alpha_{-v}v_{RB}\right)+\\ & +\frac{M_{p}}{v_{p}}\left(-\beta_{p}\frac{r_{f}p_{RB}}{1-r_{f}}-\alpha_{p}r_{f}p_{E}+\alpha_{-p}p_{RB}\right); \end{array} \end{array}$$ (28)

$$\begin{array}{c} W\frac{dl_{E}}{dt}=-\alpha_{l}r_{f}l_{E}+\alpha_{-l}l_{RB}+W\chi_{v}\rho_{v}v_{E}; \end{array}$$ (29)

$$\begin{array}{c} \frac{dl_{RB}}{dt}=\alpha_{l}r_{f}l_{E}-\alpha_{-l}l_{RB}-\beta_{l}l_{RB}; \end{array}$$ (30)

$$\begin{array}{c} \frac{dl_{I}}{dt}=\beta_{l}l_{RB}-\gamma_{l}l_{I}; \end{array}$$ (31)

$$\begin{array}{c} W\frac{dv_{E}}{dt}=-\alpha_{v}r_{f}v_{E}+\alpha_{-v}v_{RB}-W\chi_{v}v_{E}+W\omega ; \end{array}$$ (32)

$$\begin{array}{c} \frac{dv_{RB}}{dt}=\alpha_{v}r_{f}v_{E}-\alpha_{-v}v_{RB}-\beta_{v}v_{RB}; \end{array}$$ (33)

$$\begin{array}{c} \frac{dv_{I}}{dt}=\beta_{v}v_{RB}-\gamma_{v}v_{I}; \end{array}$$ (34)

$$\begin{array}{c} \frac{dc}{dt}=R_{l}^{chol}\sigma_{l}\gamma_{l}l_{I}+R_{v}^{chol}\sigma_{v}\gamma_{v}v_{I}+\mu_{c}h-\delta_{c}c; \end{array}$$ (35)

$$\begin{array}{c} W\frac{dp_{E}}{dt}=-\alpha_{p}r_{f}p_{E}+\alpha_{-p}p_{RB}+\gamma_{p}p_{I}+W\omega_{P}-\varepsilon_{p}A_{E}p_{E}+\varepsilon_{-p}p_{AB}; \end{array}$$ (36)

$$\begin{array}{c} \frac{dp_{RB}}{dt}=\alpha_{p}r_{f}p_{E}-\alpha_{-p}p_{RB}-\beta_{p}p_{RB}; \end{array}$$ (37)

$$\begin{array}{c} W\frac{dA_{E}}{dt}=-\varepsilon_{p}A_{E}p_{E}+\varepsilon_{-p}p_{AB}+W\omega_{A}; \end{array}$$ (38)

$$\begin{array}{c} \frac{dp_{AB}}{dt}=\varepsilon_{p}A_{E}p_{E}-\varepsilon_{-p}p_{AB}; \end{array}$$ (39)

$$\begin{array}{c} W\frac{dS_{E}}{dt}=-CL_{S}S_{E}+W\omega_{S}; \end{array}$$ (40)

$$\begin{array}{c} \frac{dS_{i}}{dt}=CL_{S}S_{E}-\varepsilon_{S}S_{i}h{\bar{s}}_{0}+\varepsilon_{-S}S_{ih}; \end{array}$$ (41)

$$\begin{array}{c} \frac{dS_{ih}}{dt}=\varepsilon_{S}S_{i}h{\bar{s}}_{0}-\varepsilon_{-S}S_{ih}. \end{array}$$ (42)

Sensitivity analysis was conducted for the newly introduced PCSK9-related parameters with details provided in S3 Appendix, and for the parameters associated with anti-PCSK9 agents and statins with details provided in S4 Appendix.

## Results

### Comparison with the existing model of cellular lipoprotein metabolism

**Fig 2:**
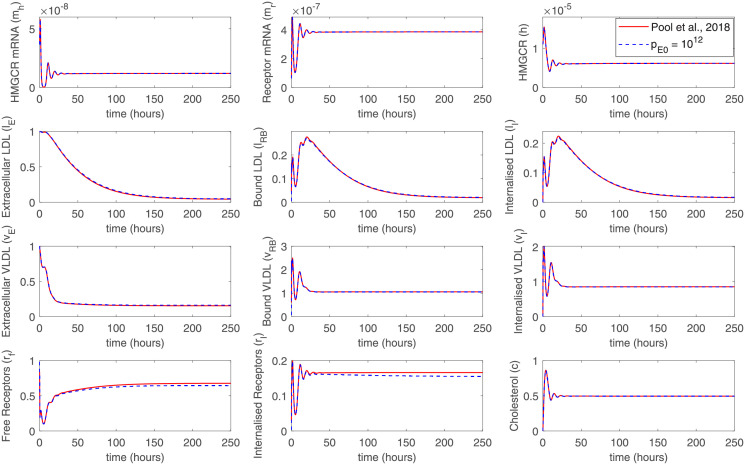
Comparison of numerical simulations conducted with the original model from [18] and with the newly developed model including PCSK9. In the latter, the concentration of PCSK9 in the extracellular space was 10^12^ molec./mL. The initial oscillations in mRNA and molecular levels, as well as the steady-state levels are reproduced well. The slow kinetics of VLDL and LDL extraction from the extracellular environment are also captured.

We have fully reproduced the original model from the [18] for the initial testing of our model. Then, the equations for the PCSK9 were introduced into the model. We can observe all the kinetic features of the original model, both at the early stages and at the steady-state (Fig 2). At first 10h, there is an increase in HMGCR, LDLR, and PCSK9 mRNA, which leads to an increase in HMGCR, internal receptors, and cholesterol. VLDL molecules have greater binding affinity and bind more rapidly than LDL to LDLR on the cell surface. Internalized LDL and VLDL provide an increase in intracellular cholesterol concentration, while receptors are released into internal storage and then recycled to the cell surface. There is no recycling for the LDLR bounded with PCSK9, but at early stages, without an external source, there is not enough PCSK9 to affect the LDLR degradation significantly. With an increase in intracellular cholesterol concentration, the transcription of HMGCR, LDLR, and PCSK9 mRNA is inhibited by negative feedback from SREBP-2. After some oscillatory type behavior, the cholesterol concentration and mRNA levels settle to a stable steady-state. A longer time is required for VLDL and LDL settling. Eventually, each component of the system settles down to a non-zero stable steady-state as a result of negative and positive feedbacks. A presence of the single steady-state was confirmed in the work [18].

### Modeling the effects of PCSK9

**Fig 3:**
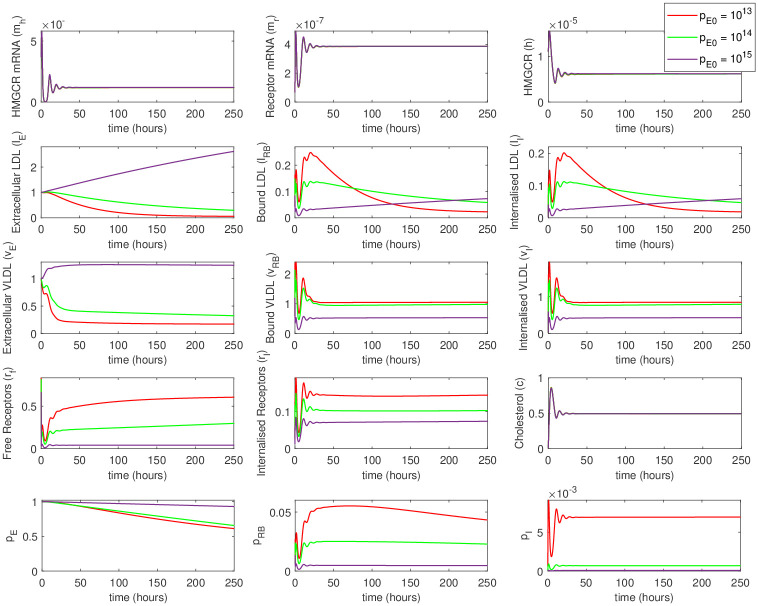
An increase in the level of PCSK9 in the extracellular space (parameter ${\bar{p}}_{E0}$) leads to an increase in the levels of LDL and VLDL, as expected in hypercholesterolemia. The effect begins at PCSK9 concentrations above 10^13^ molec./ml (hundreds of nM), that is above normal physiological concentrations.

By adjusting PCSK9 concentration in the external medium, we can reproduce the scenario of the disturbed recycling efficiency of the LDLR. PCSK9 binding to LDLR and internalization of such complex leads to the degradation of LDLR, and thus its depletion. We can directly compare the results of the simulations with varied model parameter *f*, the fraction of recycled receptors (as it was done in [18]), with the addition of different amounts of PCSK9 into the cell medium. It can be observed that specific concentration of PCSK9 capture the variations in *f*. The noticeable effect on LDLR degradation and an increase in LDL and VLDL levels was observed at concentrations of PCSK9 above 10^12^–10^13^ molec./mL (100–1000 ng/mL), which are close to physiological PCSK9 plasma level [34]. At higher initial concentrations of PCSK9 in the extracellular space (10^13^—10^14^ molec./mL) increase in LDL and VLDL levels is comparable with the effect from the direct decrease of the receptors recycling efficiency via parameter *f* (Fig 3).

**Fig 4:**
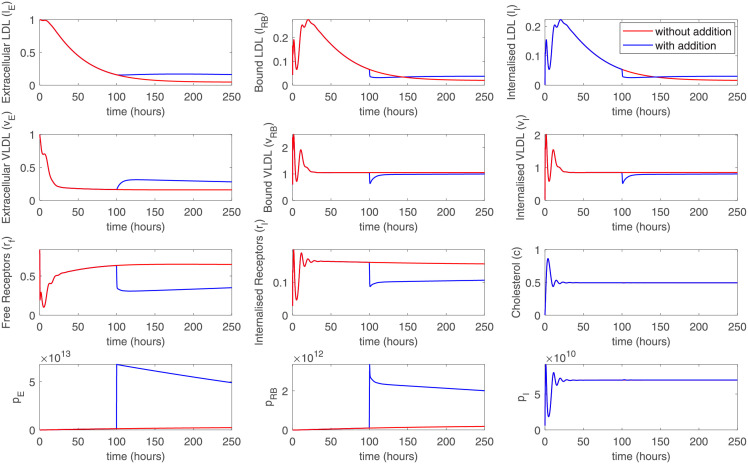
A simulation showing the effect of the addition of external PCSK9 at a predefined time point (100h). While the cells did not produce enough PCSK9 for the pronounced effects, the addition of external PCSK9 causes changes in LDL and VLDL levels.

In a particular *in vivo* experiment, it might be beneficial to add external PCSK9 molecules into the cell medium instead of waiting for its accumulation due to cellular production. We have also modeled such a situation by switching on the temporary external PCSK9 source at a selected time point (influx of 10^12^ molec./mL at 100 hours for 10 min). Thus, we can observe dynamical changes in LDL and LVDL concretions in the cell medium due to a decrease in free and internalized receptors and reduced efficiency of cellular uptake (Fig 4).

### Modeling statin therapy

**Fig 5:**
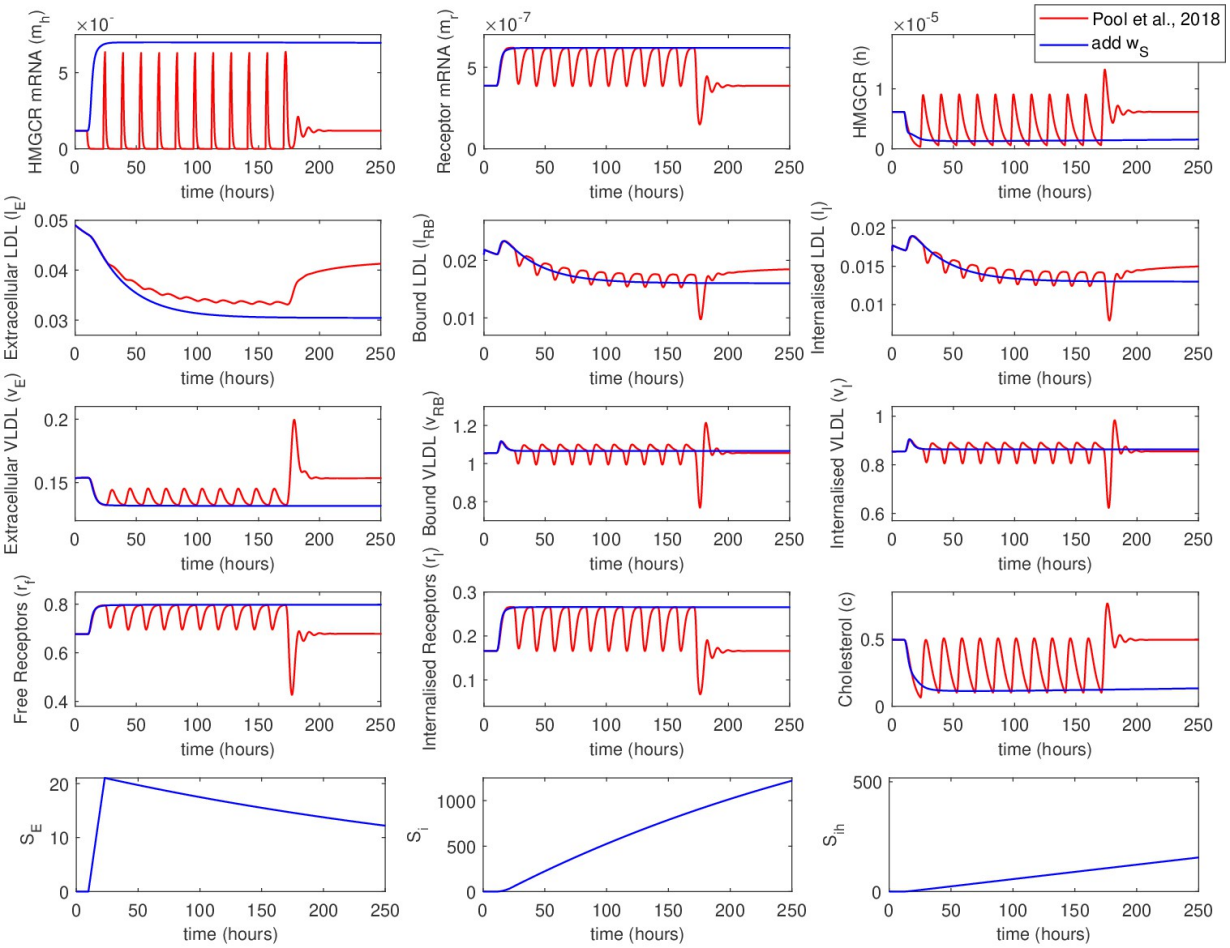
A simulation showing the effect of statin therapy in the *in vitro* experiment. Here a single dose of statin was applied at approximately *t* = 10 h. The uptake of statin by the cells is followed by inhibition of cholesterol biosynthesis by HMGCR. This also leads to up-regulation of LDLR and PCSK9 mRNA transcription, which have a competing effect on LDL and VLDL levels. Yet, a reduction caused by the LDLR is more pronounced. The numerical results for an idealized statin that instantaneously halts transcription of HMGCR mRNA (11 doses) from are also presented [18].

Statins act through inhibition of HMGCR via competitive binding and preventing its binding with HMG-CoA, thus inhibiting cholesterol biosynthesis. In the previous work, the statin therapy was modeled by modifying the transcription of HMGCR mRNA, *μ*_*mp*_, which can be considered as an idealized scenario. Here we directly modeled the stages of the statins translocation into the cell and interactions with the HMG-CoA. Statins were added into cell medium at the same time point as the first dose of “idealized” statin was applied and could reproduce a very close behavior of the system. Importantly, statins were accumulated and removed from the cell not instantly, but according to the kinetic equations. That lead to slower response of the cell, but only on the scale of minutes, and since statins were not removed from the external cell space, a single dose showed prolonged effect comparable to such effect from 11 doses over a period of 7 days (Fig 5).

### Modeling anti-PCSK9 therapy

**Fig 6:**
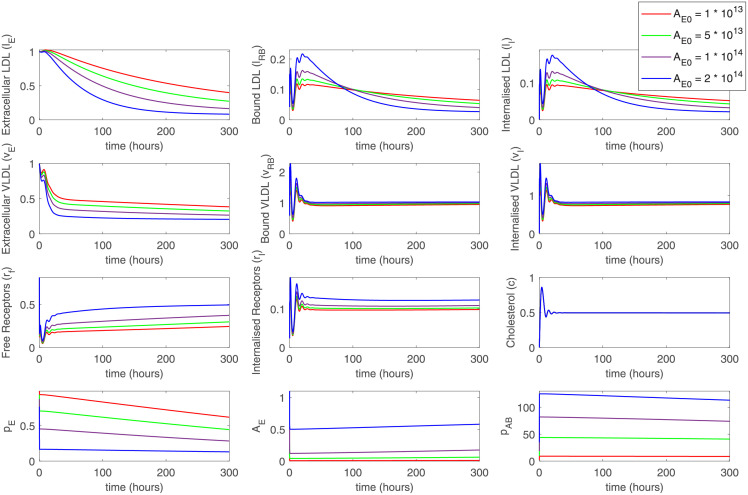
A simulation showing the effect of anti-PCSK9 antibodies therapy. Here antibodies were applied at the zero time point at different concentrations. Initial concentration of PCSK9 is 10^14^ molec./mL. The binding of PCSK9 with antibodies prevents its binding with LDLR and leads to the decrease in LDL/VLDL levels. [18].

Next, we modeled the therapy with the anti-PCSK9 agents, antibodies, or small molecules. In relation to our model, the difference between these two types of agents is purely in binding/unbinding rates ${\bar{\varepsilon }}_{p}$ and ${\bar{\varepsilon }}_{-p}$. We first consider the addition of monoclonal antibodies with typical binding/unbinding rates at zero time point simultaneously with the amount of PCSK9 that generates a high LDL level. As we can see from the comparison of graphs with and without antibodies addition, the anti-PCSK9 antibodies decrease the LDL level proportionally to the added amount (Fig 6).

**Fig 7:**
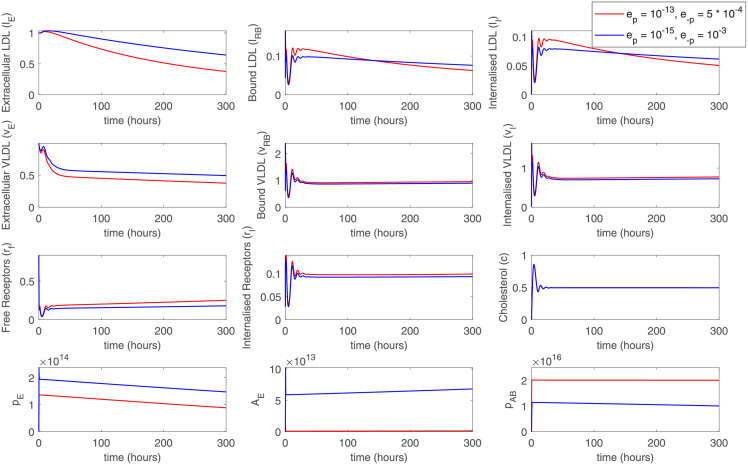
A comparison of therapies with anti-PCSK9 antibodies and small-molecule inhibitors. Due to higher association rate and lower dissociation rate, application of the inhibitory molecules (red curve) at the same concentration as antibodies (blue curve) leads to a faster and stronger decrease in the LDL level.

High cost and a requirement for injection hinder the widespread use of anti-PCSK9 monoclonal antibodies. Small-molecule inhibitors targeting protein-protein interaction of PCSK9 and LDLR were designed using special screening platforms [12, 35]. Small molecules have a larger association rates with the target than antibodies due to their smaller size and higher diffusion constant. A well-designed small-molecule inhibitor can also have a smaller dissociation rate. Taken together, that might lead to a greater inhibitory potential. By comparing antibodies with an efficient inhibitory molecule we can see a faster and more pronounced decrease in the LDL level (Fig 7).

### Modeling combined statin and anti-PCSK9 therapy

**Fig 8:**
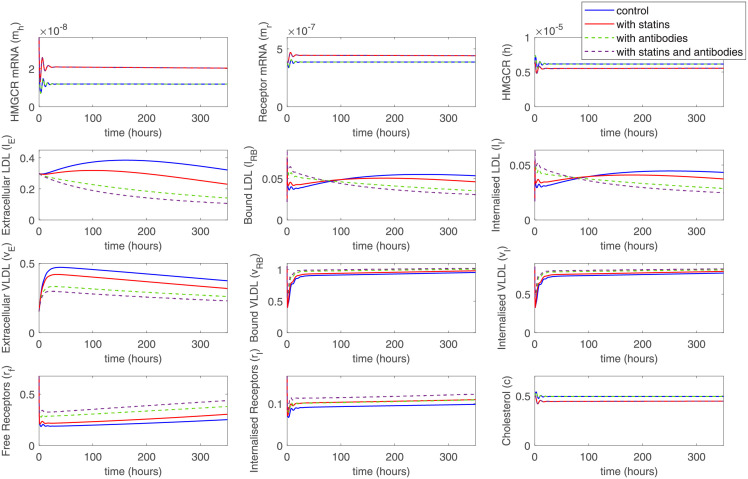
Combined therapy with statins and anti-PCSK9 agents might demonstrate additive effects on LDL and VLDL levels.

We have also modeled a combined therapy with the use of statins and anti-PCSK9 agents. As it was shown in experiments with dyslipidemic rhesus monkeys, such combined therapy provides a more substantial reduction in LDL level than either agent alone [36]. An additive effect was reproduced here by more substantial decrease in extracellular LDL and VLDL then anti-PCSK9 antibodies and statins were introduced into the system simultaneously (Fig 8).

## Discussion and conclusion

In the current work, we have formulated, conducted simulations, and analyzed a nonlinear deterministic ODE model of the key processes related to cholesterol metabolism in hepatocytes. The model is based on the previously published model, where the key mechanisms of hepatocyte endocytosis of VLDL and LDL coupled with cholesterol and LDLR biosynthesis via the HMGCR pathway were addressed [18]. The presented model provides several improvements over the existing one. First, we have included PCSK9 and its effects in the model. Second, anti-PCSK9 and statin therapies were modeled for an *in vitro* scenario. PCSK9 is an important component that affects cholesterol metabolism at the organism level [7, 9]. A higher circulating PCSK9 level was shown to be associated with incident cardiovascular events [37, 38]. Importantly, PCSK9 is regulated by the same protein SREBP2 as the LDLR and HMGCR [39, 40]. Therefore, statin treatment leads to elevated expression of PCSK9 that might have a negative effect and reduce the overall treatment efficiency [7, 11]. It should be noted, however, that statins may exert cardiovascular protective effects that are independent of LDL lowering. These are called pleiotropic effects and include alterations in the expression of endothelial nitric oxide synthase, the stability of atherosclerotic plaques, the production of proinflammatory cytokines and reactive oxygen species, the reactivity of platelets, and others [10, 41]. Such effects might contribute to clinical outcomes, yet it is impossible to include them in the current model.

Anti-PCSK9 therapy is an intensively developing field with multiple introduced therapeutic modalities, including antibodies, small molecules, and small interfering RNA [7, 19, 35]. The mathematical model can potentially benchmark the efficiency of different therapy types, as it was done in [19]. Such a comparison using our model predicts a higher efficiency of small-molecule inhibitor of PCSK9 binding with LDLR over traditionally used antibodies. However, further *in vitro* and *in vivo* tests are required to confirm this prediction.

As shown above, the presented model can partially reproduce the metabolism of the cholesterol *in vivo*, including some clinically observed effects of hyperlipidemia and its treatment. However, the model does not contain a detailed description of some significant elements of lipoprotein metabolism, which are important *in vivo*. These are, for example, the transport of dietary lipids from the intestines via chylomicrons, production of VLDL by liver cells, intermediate and high-density lipoprotein fractions, and enterohepatic cholesterol circulation with bile. Furthermore, *in vivo* measurements and clinical trials examine longer time intervals, weeks or months. The existing *in vivo* models, however, are also limited by an only partial representation of the compartments (e.g., the single plasma compartment [19], or two compartments of liver and plasma [21, 22]), or imply great simplification of molecular aspects [23, 42]. In principle, the model presented here can be adapted for reuse within a hierarchy of the larger whole-body model [43], although its current primary purpose is description and prediction of *in vitro* tests.

Since the presented model describes mostly the cellular and subcellular processes that occur during lipoprotein metabolism, it is suited most for *in vitro* experiments. Although *in vitro* experiments lack the complex environment of a multi-cellular organism and cannot predict the biokinetic profile of a tested chemical, it is an important stage of drug design and testing. These models use homogeneous and characterized cells, are economic and easily replicated, lack concerns on animal testing, and provide mechanistic data which can be directly compared with the mathematical models [44]. Recent developments in *in vitro* screening platforms, such as the use of induced pluripotent stem cells (iPSC), iPSC-derived organoids, organ-on-a-chip, and multi-organ-on-a-chip models allow personalized testing of drug safety and efficacy [24], while keeping the complexity of the system within limits that could be effectively modeled with mechanistic models.

To conclude, here we have improved the existing model of the cellular and subcellular processes that occur during lipoprotein metabolism by including the effects of PCSK9. In a single mechanistic model, it is now possible to simulate the effects of two types of hyperlipidemia treatment, namely with statins and anti-PCSK9 agents. The efficiency of different treatment strategies can be directly compared, and a higher effect of a small-molecule inhibitor of PCSK9 over antibodies was observed in the simulation, as well as the additive effect of combined therapy with statins and anti-PCSK9 agents. Therefore, the model can be useful at the stage of the *in vitro* testing of the drugs, where both model parameters and experiment setup can be adjusted to achieve the best discrimination of the drug efficiency.

## Supporting information

S1 Appendix Description of the model parameters. (PDF)

S2 Appendix Non-Dimensionalisation of the model. (PDF)

S3 Appendix Sensitivity analysis for the PCSK9-included model. (PDF)

S4 Appendix Sensitivity analysis for the antibody and statin treatments. (PDF)
