# Supplementary material for: A mathematical model of in vitro hepatocellular cholesterol and lipoprotein metabolism for hyperlipidemia therapy
Source: PLoS One. 2022 Jun 3;17(6):e0264903. doi: 10.1371/journal.pone.0264903 (PMC9165868; doi:10.1371/journal.pone.0264903)
Supplement: S1 Appendix — (PDF) [file pone.0264903.s001.pdf]

S1 Appendix. Description of the model parameters. The newly introduced parameters are indicated in bold.

Parameter	Description	Parameter	Description
$\bar{\mu}^*_{mh}$	Rate of HMGCR mRNA transcription	$\bar{\mu}^*_{mr}$	Rate of receptor mRNA transcription
$\bar{\mu}^*_{mp}$	**Rate of PCSK9 mRNA transcription**	$\bar{\mu}_h$	Rate of HMGCR translation
$\bar{\mu}_r$	Rate of LDLR translation	$\bar{\mu}_c$	Rate of cholesterol production
$\bar{\mu}_p$	**Rate of PCSK9 translation**	$\bar{\delta}_{mh}$	Rate of HMGCR mRNA degradation
$\bar{\delta}_{mr}$	Rate of LDLR mRNA degradation	$\bar{\delta}_{mp}$	**Rate of PCSK9 mRNA degradation**
$\bar{\delta}_h$	Rate of HMGCR degradation	$\bar{\delta}_c$	Rate of cholesterol degradation
$\bar{\delta}_p$	**Rate of intracellular PCSK9 degradation**	x_h	Number of binding sites for SREBP-2 on HMGCR gene
x_r	Number of binding sites for SREBP-2 on LDLR gene	x_p	**Number of binding sites for SREBP-2 on PCSK9 gene**
x_c	Molec. of cholesterol to inactivate SREBP-2	$\bar{k}_{mh}$	HMGCR gene-SREBP-2 binding affinity
$\bar{k}_{mr}$	LDLR gene-SREBP-2 binding affinity	$\bar{k}_{mp}$	**PCSK9 gene-SREBP-2 binding affinity**
$\bar{k}_c$	Cholesterol-SREBP-2 dissociation constant	$\bar{\gamma}_p$	**Rate of PCSK9 transport to the extracellular space**
$\bar{\alpha}_l$	Rate of LDL-receptor binding	$\bar{\alpha}_{-l}$	Rate of LDL-receptor unbinding
$\bar{\alpha}_v$	Rate of VLDL-receptor binding	$\bar{\alpha}_{-v}$	Rate of VLDL-receptor unbinding
$\bar{\alpha}_p$	**Rate of PCSK9-receptor binding**	$\bar{\alpha}_{-p}$	**Rate of PCSK9-receptor unbinding**
M_l	Receptors covered by bound LDL	M_v	Receptors covered by bound VLDL
M_p	**Receptors covered by bound PCSK9**	P	Number of receptors per pit
f	Fraction of receptors recycled	R_l^{chol}	Average cholesterol content per LDL
R_v^{chol}	Average cholesterol content per VLDL	$\bar{\beta}_l$	Rate of LDL internalisation
$\bar{\beta}_v$	Rate of VLDL internalisation	$\bar{\beta}_0$	Rate of free receptor internalisation
$\bar{\beta}_p$	**Rate of PCSK9 internalisation**	$\bar{\gamma}_l$	Rate of LDL to cholesterol conversion
$\bar{\gamma}_v$	Rate of VLDL to cholesterol conversion	$\bar{\gamma}_r$	Rate of receptor recycling
$\bar{\chi}_v$	Rate of VLDL-LDL delipidation	$\bar{\varepsilon}_S$	**Rate of Statin-HMGCR binding**
$\bar{\varepsilon}_{-S}$	**Rate of Statin-HMGCR unbinding**	$\bar{CL}_S$	**Cell uptake clearance of statins**
$\bar{\varepsilon}_p$	**Rate of antibody-PCSK9 binding**	$\bar{\varepsilon}_{-p}$	**Rate of antibody-PCSK9 unbinding**
J	Nucleus to cell ratio	W	Cell medium to cell volume ratio
$\bar{\omega}$	Influx of extracellular VLDL	$\bar{\omega}_P$	**Influx of extracellular PCSK9**
$\bar{\omega}_A$	**Influx of anti-PCSK9 agents**	$\bar{\omega}_S$	**Influx of statins**

State variables

$\bar{m}_h$	HMGCR mRNA concentration
$\bar{m}_r$	LDLR mRNA concentration
$\bar{m}_p$	**PCSK9 mRNA concentration**
$\bar{h}$	**HMGCR concentration**
$\bar{c}$	Intracellular cholesterol concentration
$\bar{r}_I, \bar{r}_f$	Internalised and unbound receptor concentrations
$\bar{l}_E, \bar{l}_{RB}, \bar{l}_I$	Unbound extracellular, receptor-bound and internalised LDL concentrations
$\bar{v}_E, \bar{v}_{RB}, \bar{v}_I$	Unbound extracellular, receptor-bound and internalised VLDL concentrations
$\bar{p}_E, \bar{p}_{RB}, \bar{p}_{AB}, \bar{p}_I$	**Unbound extracellular, receptor-bound, anti-PCSk9 agent-bound and internalised PCSK9 concentrations**
$\bar{A}_E$	**Unbound extracellular anti-PCSk9 agent concentration**
$\bar{S}_E, \bar{S}_i, \bar{S}_{ih}$	**Unbound extracellular, free internalised, HMGCR-bound statin concentrations**
