# Supplementary material for: A mathematical model of in vitro hepatocellular cholesterol and lipoprotein metabolism for hyperlipidemia therapy
Source: PLoS One. 2022 Jun 3;17(6):e0264903. doi: 10.1371/journal.pone.0264903 (PMC9165868; doi:10.1371/journal.pone.0264903)
Supplement: S2 Appendix — (PDF) [file pone.0264903.s002.pdf]

S2 Appendix. Non-Dimensionalisation of the model. The following rescalings from the work [18] were used:

$$\bar{t} = \frac{t}{\bar{\delta}_{mh}}, \qquad \bar{m}_h = \bar{s}_0 m_h, \qquad \bar{m}_r = \bar{s}_0 m_r, \qquad \bar{h} = \bar{s}_0 h, \qquad \bar{r}_I = \bar{r}_{f0} r_I,$$

$$\bar{r}_f = \bar{r}_{f0} r_f, \qquad \bar{l}_E = \bar{l}_{E0} l_E, \qquad \bar{l}_{RB} = \bar{l}_{E0} l_{RB}, \qquad \bar{l}_I = \bar{l}_{E0} l_I, \qquad \bar{v}_E = \bar{v}_{E0} v_E,$$

$$\bar{v}_{RB} = \bar{v}_{E0} v_{RB}, \qquad \bar{v}_I = \bar{v}_{E0} v_I, \qquad \bar{c} = \bar{c}_0 c.$$

The rescalings for the newly introduced parameters are:

$$\bar{m}_p = \bar{s}_0 m_p, \qquad \bar{p}_I = \bar{p}_{E0} p_I, \qquad \bar{p}_E = \bar{p}_{E0} p_E,$$

$$\bar{p}_{RB} = \bar{p}_{E0} p_{RB}, \qquad \bar{A}_E = \bar{p}_{E0} A_E, \qquad \bar{p}_{AB} = \bar{p}_{E0} p_{AB},$$

$$\bar{S}_E = \bar{S}_{E0} S_E, \qquad \bar{S}_i = \bar{S}_{E0} S_i, \qquad \bar{S}_{ih} = \bar{S}_{E0} S_{ih}.$$

Parameters used for the rescalings

**Parameter**	**Description**	**Value**	**Units**
$\bar{c}_0$	Total concentration of cholesterol in a hepatocyte	1.89×10^{19}	$\frac{molec.}{mL}$
$\bar{s}_0$	Total concentration of SREBP-2 in a hepatocyte	8.21×10^{16}	$\frac{molec.}{mL}$
$\bar{r}_{f0}$	Initial unbound receptor concentration	3.27×10^{13}	$\frac{molec.}{mL}$
$\bar{l}_{E0}$	Initial extracellular LDL concentration	1.17×10^{13}	$\frac{molec.}{mL}$
$\bar{v}_{E0}$	Initial extracellular VLDL concentration	2.95×10^{12}	$\frac{molec.}{mL}$
$\bar{\delta}_{mh}$	Rate of HMGCR mRNA degradation	4.48×10^{-5}	$\frac{1}{s}$
$\bar{p}_{E0}$	Initial extracellular PCSK9 concentration	10^{14}	$\frac{molec.}{mL}$
$\bar{S}_{E0}$	Initial extracellular statins concentration	10^{12}	$\frac{molec.}{mL}$
