# Supplementary material for: A mathematical model of in vitro hepatocellular cholesterol and lipoprotein metabolism for hyperlipidemia therapy
Source: PLoS One. 2022 Jun 3;17(6):e0264903. doi: 10.1371/journal.pone.0264903 (PMC9165868; doi:10.1371/journal.pone.0264903)
Supplement: S3 Appendix — (PDF) [file pone.0264903.s003.pdf]

S3 Appendix. Sensitivity analysis for the PCSK9-included model. Here we presented the results of the sensitivity analysis for the newly introduced parameters showing the relative percentage change from steady-state as each parameter was varied within indicated range (generally 100-fold below and above the nominal values, which are indicated in the first cell under the parameter). Some columns are not shown due to low change in values (HMGCR and LDLR mRNA, HMGCR, cholesterol). "LE" is extracellular LDL, "LRB" is receptor bound LDL, "LI" is internalised LDL, "VE" is extracellular VLDL, "VRB" is receptor bound VLDL, "VI" is internalised VLDL, "RF" is free receptors, "RI" is internalised receptors, "mRNA P" is PCSK9 mRNA, "PE" is extracellular PCSK9, "PRB" is receptor bound PCSK9, "PI" is internalised PCSK9.
The following color code is used: green indicates a change below 10%, yellow in a range 10% - 50%, and pink above 50% relative to the steady-state value.

		Percent difference in											
**Parameter**	**Value**	**LE**	**LRB**	**LI**	**VE**	**VRB**	**VI**	**RF**	**RI**	**mRNA P**	**PI**	**PE**	**PRB**
μ_{mp} **2,72E-07**	2.72E-05	750.60	45.02	44.99	262.47	-38.18	-38.18	-82.95	-27.89	9914.33	9914.33	1170.37	116.60
	2.72E-06	73.09	19.44	19.43	36.98	-5.47	-5.47	-30.99	-9.11	900.09	900.09	96.39	35.51
	2.72E-08	-6.77	-2.51	-2.51	-3.83	0.56	0.56	4.57	1.50	-90.00	-90.00	-9.19	-5.04
	2.72E-09	-7.44	-2.77	-2.77	-4.21	0.62	0.62	5.04	1.66	-99.00	-99.00	-10.11	-5.57
μ_p **1,83E+06**	1.83E+08	751.47	45.08	45.05	262.59	-38.20	-38.19	-82.96	-27.89	0.14	9914.34	1171.20	116.62
	1.83E+07	73.16	19.47	19.46	37.00	-5.47	-5.47	-31.00	-9.11	0.01	900.09	96.45	35.53
	1.83E+05	-6.78	-2.52	-2.51	-3.83	0.56	0.56	4.57	1.50	0.00	-90.00	-9.20	-5.05
	1.83E+04	-7.45	-2.77	-2.77	-4.21	0.62	0.62	5.05	1.66	0.00	-99.00	-10.11	-5.57
x_p **1,00E+00**	1.00E+02	-7.50	-2.79	-2.79	-4.25	0.63	0.63	5.09	1.67	-100.00	-100.00	-10.19	-5.62
	1.00E+01	-7.48	-2.78	-2.78	-4.24	0.62	0.62	5.08	1.67	-99.89	-99.89	-10.17	-5.61
	1.00E-01	4.25	1.51	1.51	2.34	-0.34	-0.34	-2.63	-0.83	54.20	54.20	5.69	2.91
	1.00E-02	4.71	1.67	1.67	2.59	-0.38	-0.38	-2.90	-0.92	59.93	59.93	6.30	3.22
m_p **1,00E+00**	1.00E+02	3493.08	-51.15	-51.17	650.13	-89.80	-89.80	-98.64	-48.40	0.48	0.48	110.67	-97.14
	1.00E+01	1623.41	103.94	103.88	343.13	-47.55	-47.55	-88.16	-29.81	0.13	0.13	90.73	-77.43
	1.00E-01	-75.64	-54.25	-54.25	-43.76	5.61	5.61	87.80	47.26	0.02	0.02	-56.23	-17.78
	1.00E-02	-77.67	-56.46	-56.47	-45.72	5.85	5.85	94.98	55.01	0.02	0.02	-61.53	-24.98
γ_p **2,23E+02**	2.23E+04	0.00	0.00	0.00	0.00	0.00	0.00	0.00	0.00	0.00	-99.00	0.00	0.00
	2.23E+03	0.00	0.00	0.00	0.00	0.00	0.00	0.00	0.00	0.00	-90.00	0.00	0.00
	2.23E+01	-0.01	0.00	0.00	0.00	0.00	0.00	0.00	0.00	0.00	900.00	-0.01	0.00
	2.23E+00	-0.07	-0.04	-0.04	-0.03	0.00	0.00	0.03	0.01	0.00	9899.97	-0.06	-0.03
α_p **7,30E+00**	7.30E+02	1001.32	1046.70	1047.30	-1.75	2.24	2.24	4.07	46.89	-0.95	-0.95	-99.88	-87.04
	7.30E+01	649.47	323.80	323.66	67.85	-5.07	-5.08	-43.43	-1.04	-0.28	-0.28	-78.06	24.17
	7.30E-01	-69.63	-49.40	-49.41	-37.19	4.68	4.68	66.65	29.44	0.02	0.02	83.67	-69.39
	7.30E-02	-76.99	-55.86	-55.87	-44.90	5.73	5.73	91.88	51.47	0.02	0.02	109.75	-95.98
α_{-p} **2,23E+00**	2.23E+02	-60.42	-42.02	-42.03	-29.26	3.63	3.63	46.50	17.45	0.02	0.02	60.55	-48.15
	2.23E+01	-19.76	-13.01	-13.01	-7.00	0.83	0.83	8.41	2.34	0.01	0.01	11.60	-8.44
	2.23E-01	2.77	1.78	1.78	0.87	-0.10	-0.10	-0.96	-0.25	0.00	0.00	-1.34	0.95
	2.23E-02	3.06	1.96	1.96	0.96	-0.11	-0.11	-1.06	-0.27	0.00	0.00	-1.48	1.05
β_p **6,03E+01**	6.03E+03	3.09	1.99	1.99	0.97	-0.11	-0.11	-1.07	-0.27	0.00	0.00	-1.46	-98.99
	6.03E+02	2.79	1.79	1.79	0.88	-0.10	-0.10	-0.97	-0.25	0.00	0.00	-1.33	-89.91
	6.03E+00	-19.78	-13.03	-13.03	-7.01	0.83	0.83	8.43	2.35	0.01	0.01	11.56	815.98
	6.03E-01	-60.52	-42.09	-42.10	-29.36	3.64	3.64	46.71	17.56	0.02	0.02	59.84	5075.14
δ_{mp} **1,00E+00**	1.00E+02	-7.45	-2.77	-2.77	-4.21	0.62	0.62	5.05	1.66	-99.00	-99.00	-10.11	-5.57
	1.00E+01	-6.78	-2.52	-2.51	-3.83	0.56	0.56	4.57	1.50	-90.00	-90.00	-9.20	-5.05
	1.00E-01	71.50	18.89	18.88	36.45	-5.40	-5.40	-30.67	-9.04	900.08	900.08	94.98	35.16
	1.00E-02	579.29	33.36	33.34	233.70	-34.47	-34.46	-80.36	-26.71	9888.43	9888.42	977.27	111.51
k_{mp} **1,00E+00**	1.00E+02	-7.41	-2.76	-2.76	-4.19	0.62	0.62	5.02	1.65	-98.55	-98.55	-10.06	-5.54
	1.00E+01	-6.48	-2.40	-2.40	-3.66	0.54	0.54	4.36	1.43	-86.11	-86.11	-8.80	-4.82
	1.00E-01	12.68	4.30	4.30	6.93	-1.02	-1.02	-7.43	-2.33	162.98	162.98	16.99	8.29
	1.00E-02	16.85	5.63	5.62	9.13	-1.35	-1.35	-9.60	-2.98	214.18	214.18	22.49	10.72
$\bar{p}_{E0}$ **1,00E+14**	1.00E+16	3479.72	-48.41	-48.43	646.39	-89.24	-89.24	-98.56	-48.14	0.47	-99.00	98.18	-97.14
	1.00E+15	1587.97	109.95	109.89	333.38	-46.08	-46.08	-87.56	-29.10	0.12	-89.99	79.53	-77.67
	1.00E+13	-73.27	-52.24	-52.25	-41.10	5.24	5.24	78.66	38.74	0.02	900.22	0.05	78.75
	1.00E+12	-75.36	-54.44	-54.45	-42.98	5.46	5.46	84.94	44.33	0.02	9902.28	513.70	1034.78
	1.00E+11	-75.54	-54.63	-54.63	-43.14	5.48	5.48	85.51	44.87	0.02	99922.88	5701.37	10659.69
