# Supplementary material for: A mathematical model of in vitro hepatocellular cholesterol and lipoprotein metabolism for hyperlipidemia therapy
Source: PLoS One. 2022 Jun 3;17(6):e0264903. doi: 10.1371/journal.pone.0264903 (PMC9165868; doi:10.1371/journal.pone.0264903)
Supplement: S4 Appendix — (PDF) [file pone.0264903.s004.pdf]

S4 Appendix. Sensitivity analysis for the antibody and statin treatments. The additional sensitivity analysis performed for the parameters associated with the antibody and statin treatments. Here "mRNA H" is HMGCR mRNA, "AE" is extracellular antibodies, "PAB" is PCSK9 bound antibodies, "SE" is extracellular statins, "SI" is internalised statins, "SIH" is HMGCR bound statins. The nominal values are indicated in the first cell under the parameter.

		Percent difference in																				
**Parameter**	**Value**	**mRNA H**	**mRNA LDLR**	**HMGCR**	**LE**	**LRB**	**LI**	**VE**	**VRB**	**VI**	**RF**	**RI**	**Cholesterol**	**mRNA P**	**PI**	**PE**	**PRB**	**AE**	**PAB**	**SE**	**SI**	**SIH**
ε_p **2,23E-10**	2.23E-08	0.01	0.00	0.01	-20.92	-10.04	-10.04	-10.60	1.70	1.70	13.76	3.65	0.00	0.00	0.00	-22.28	-11.59	-98.42	22.94	0.00	0.00	0.00
	2.23E-09	0.01	0.00	0.01	-18.34	-8.80	-8.80	-9.15	1.46	1.46	11.69	3.07	0.00	0.00	0.00	-19.27	-9.83	-85.15	19.85	0.00	0.00	0.00
	2.23E-11	-0.03	-0.01	-0.03	60.23	25.28	25.27	23.28	-3.61	-3.61	-21.81	-4.98	0.01	-0.01	-0.01	51.37	18.35	220.72	-51.45	0.00	0.00	0.00
	2.23E-12	-0.06	-0.02	-0.06	117.59	45.10	45.09	40.60	-6.24	-6.24	-33.31	-7.31	0.01	-0.02	-0.02	92.09	28.09	388.75	-90.61	0.00	0.00	0.00
ε_{-p} **2,23E+01**	2.23E+03	-0.06	-0.02	-0.06	117.59	45.10	45.09	40.60	-6.24	-6.24	-33.31	-7.31	0.01	-0.02	-0.02	92.09	28.09	388.77	-90.61	0.00	0.00	0.00
	2.23E+02	-0.03	-0.01	-0.03	60.23	25.27	25.27	23.29	-3.61	-3.61	-21.81	-4.98	0.01	-0.01	-0.01	51.38	18.35	220.74	-51.45	0.00	0.00	0.00
	2.23E+00	0.01	0.00	0.01	-18.34	-8.79	-8.79	-9.15	1.46	1.46	11.69	3.07	0.00	0.00	0.00	-19.28	-9.84	-85.15	19.85	0.00	0.00	0.00
	2.23E-01	0.01	0.00	0.01	-20.92	-10.04	-10.03	-10.60	1.70	1.70	13.76	3.66	0.00	0.00	0.00	-22.29	-11.59	-98.42	22.94	0.00	0.00	0.00
$\bar{A}_{E0}$ **1,00E+13**	1.00E+15	0.14	0.03	0.14	-85.74	-65.32	-65.33	-55.04	9.34	9.34	143.19	55.44	-0.02	0.03	0.03	-96.76	-92.13	0.00	0.00	0.00	0.00	0.00
	1.00E+14	0.09	0.02	0.09	-58.46	-38.11	-38.11	-29.52	5.02	5.02	49.00	11.12	-0.01	0.02	0.02	-54.03	-31.50	0.00	0.00	0.00	0.00	0.00
	1.00E+12	-0.01	0.00	-0.01	8.92	4.25	4.25	3.79	-0.66	-0.66	-4.29	-0.84	0.00	0.00	0.00	7.45	2.84	0.00	0.00	0.00	0.00	0.00
	1.00E+11	-0.01	0.00	-0.01	9.84	4.67	4.67	4.17	-0.73	-0.73	-4.71	-0.92	0.00	0.00	0.00	8.21	3.12	0.00	0.00	0.00	0.00	0.00
ε_S **2,23E-10**	2.23E-08	14.64	3.48	-2.44	-9.38	-4.61	-4.61	-4.26	0.77	0.77	5.26	2.79	-2.39	3.48	3.48	-2.58	2.55	0.00	0.00	0.00	-96.98	194.86
	2.23E-09	11.25	2.70	-1.90	-7.40	-3.61	-3.61	-3.35	0.61	0.61	4.09	2.17	-1.85	2.70	2.70	-2.01	1.99	0.00	0.00	0.00	-74.54	149.77
	2.23E-11	-6.38	-1.64	1.15	4.90	2.29	2.29	2.15	-0.39	-0.39	-2.49	-1.31	1.12	-1.64	-1.64	1.25	-1.26	0.00	0.00	0.00	42.59	-85.58
	2.23E-12	-7.34	-1.89	1.33	5.69	2.65	2.65	2.49	-0.45	-0.45	-2.87	-1.51	1.30	-1.89	-1.89	1.45	-1.46	0.00	0.00	0.00	49.02	-98.49
ε_{-S} **2,23E+02**	2.23E+04	-7.34	-1.89	1.33	5.69	2.65	2.65	2.49	-0.45	-0.45	-2.87	-1.51	1.30	-1.89	-1.89	1.45	-1.46	0.00	0.00	0.00	49.02	-98.49
	2.23E+03	-6.38	-1.64	1.15	4.90	2.29	2.29	2.15	-0.39	-0.39	-2.49	-1.31	1.12	-1.64	-1.64	1.25	-1.26	0.00	0.00	0.00	42.59	-85.58
	2.23E+01	11.25	2.70	-1.90	-7.40	-3.61	-3.61	-3.35	0.61	0.61	4.09	2.17	-1.85	2.70	2.70	-2.01	1.99	0.00	0.00	0.00	-74.53	149.75
	2.23E+00	14.64	3.48	-2.44	-9.38	-4.61	-4.61	-4.26	0.77	0.77	5.26	2.79	-2.39	3.48	3.48	-2.58	2.55	0.00	0.00	0.00	-96.96	194.82
CL_S **2,23E-01**	2.23E+01	-6.79	-1.75	1.29	-16.50	-12.60	-12.61	-3.84	0.65	0.66	4.68	-0.43	1.20	-1.75	-1.75	-9.49	-5.26	0.00	0.00	-99.99	1048.36	1063.29
	2.23E+00	26.62	6.08	-4.23	-20.93	-12.20	-12.20	-8.60	1.49	1.49	11.04	5.09	-4.17	6.08	6.08	-6.93	3.35	0.00	0.00	-55.79	597.49	568.03
	2.23E-02	-6.64	-1.71	1.20	5.13	2.40	2.40	2.25	-0.41	-0.41	-2.60	-1.36	1.17	-1.71	-1.71	1.31	-1.32	0.00	0.00	8.50	-89.63	-89.50
	2.23E-03	-7.37	-1.90	1.33	5.72	2.67	2.67	2.50	-0.45	-0.45	-2.88	-1.52	1.30	-1.90	-1.90	1.46	-1.47	0.00	0.00	9.39	-98.96	-98.95
$\bar{S}_{E0}$ **1,00E+12**	1.00E+14	412.48	54.19	-57.49	-67.93	-43.91	-43.91	-38.83	6.97	6.97	74.88	46.15	-56.05	54.19	54.19	-30.15	22.18	0.00	0.00	0.00	23.62	-47.45
	1.00E+13	64.34	13.24	-9.42	-29.91	-15.96	-15.95	-14.43	2.61	2.61	19.91	10.76	-9.20	13.24	13.24	-9.37	8.68	0.00	0.00	0.00	3.23	-6.49
	1.00E+11	-6.71	-1.72	1.21	5.16	2.41	2.41	2.27	-0.41	-0.41	-2.62	-1.38	1.18	-1.72	-1.72	1.32	-1.33	0.00	0.00	0.00	-0.40	0.80
	1.00E+10	-7.38	-1.90	1.33	5.72	2.67	2.67	2.51	-0.45	-0.45	-2.89	-1.52	1.30	-1.90	-1.90	1.46	-1.47	0.00	0.00	0.00	-0.44	0.89
